# Study about incidence of nosocomial infections in elderly care institutions and control measures

**DOI:** 10.1186/1753-6561-5-S6-P159

**Published:** 2011-06-29

**Authors:** AM Costa E Silva, ACDSD Oliveira, S Scota, ES Abreu, ILA França e Silva

**Affiliations:** 1 Hospitalar Infection Control Comission, Instituto de Infectologia Emìlio Ribas, São Paulo, Brazil

## Introduction / objectives

Infections in the elderly are the major cause of morbidity, mortality and hospitalization, beyond the difficulty of approach and diagnosis. The objective estimate the incidence of nosocomial infections in the institution.

## Methods

The study was performed in a hospital specialized in care of chronic patients with high dependency, in special elderly, advanced-stage cancer patients, dementia, severe neurological deficit and other chronic diseases. It holds 77 beds in three internment units, a semi-intensive care unit and emergency care. Data were collected daily through audit, and as the Brazilian statistics are being stablished, international data of incidence/patients-day were used for comparison.

## Results

Rates of Hospitalar Infection in the institution were:

· General Rate of Hospitalar Infection: 10.31 infections/1000 patients-day

· Pneumonia Rate: 3.78 infections/1000 patients-day

· Urinary Tract Infection Rate: 4.12 infections/1000 patients-day

We carried out control measures aiming at prevention of urinary tract infection, such as clear-sighted evaluation of real need of bladder catheterization, nursing staff training regarding the technique and careful maintenance of bladder catheterization, which also included simple care such as using individual container for each patient to discard the urine. Enhanced training for hand hygiene. After thirty days of intervention there was reduction in the rate of Urinary Tract Infection from 4.12 infections/1000 patients-day to 3.51 infections/1000 patients-day.

## Conclusion

Ideal level wasn't yet achieved, but we believe that with the continuous training of care during introduction, maintenance and clear-sighted evaluation for catheterization, lower rates of infection will be achieved.

## Disclosure of interest

None declared.

